# Prediction of Bending Mechanical Behaviors of SiC_f_/SiC 2.5D Woven Composites with Random Pore Defects

**DOI:** 10.3390/ma19050934

**Published:** 2026-02-28

**Authors:** Xiaomeng Wang, Tiantian Yang, Ling Wang, Weijie Xie, Kun Qian, Mingwei Chen, Haipeng Qiu, Diantang Zhang

**Affiliations:** 1 AVIC Composite Technology Center, AVIC Manufacturing Technology Institute, Beijing 101300, China; bradywang01@163.com (X.W.); lingwang14802@163.com (L.W.); 18810536240@126.com (W.X.); mingwei070806@163.com (M.C.); 2 Department of Astronautic Science and Mechanics, Harbin Institute of Technology, Harbin 150001, China; 22b318013@stu.hit.edu.cn; 3 Key Laboratory of Special Protective Textiles, Ministry of Education, College of Textile Science and Engineering, Jiangnan University, Wuxi 214122, China; qiankun_8@163.com

**Keywords:** 2.5D woven reinforcement, SiC_f_/SiC composites, Micro-CT analysis, three-point bending, pore defects, numerical simulations

## Abstract

The inevitable pore defects generated in the preparation process have a great impact on the mechanical properties of the ceramic matrix composites. However, the pore defects on the composites were ignored to a large extent in models established in the previous research. In this study, in order to investigate the bending damage behaviors of SiC_f_/SiC (SiC fiber-reinforced SiC matrix) angle-interlock (2.5D) woven composites prepared by the precursor immersion pyrolysis (PIP) method, a more precise full-scale model of composites was established by finite element (FE) method with taking into account of random pore defects generated by Monte Carlo algorithm. Micro-computed tomography (Micro-CT) was employed to acquire the statistical data of the yarns and pores of SiC_f_/SiC 2.5D woven composites. A bending test was conducted to study the damage behaviors of the composite and compared with the prediction of the FE model. The result shows that the proposed model with random pores can predict the mechanical damage behavior of SiC_f_/SiC 2.5D woven composites effectively under three-point bending. The simulated bending strength shows a good agreement with the experimental data, with a relative error of approximately 4.6%.

## 1. Introduction

Silicon carbide (SiC)-based materials exhibit immense potential across various high-tech fields. For instance, Huczko et al. [1] highlighted the synthesis and cathodoluminescence properties of SiC nanowires, suggesting applications in optoelectronics. Furthermore, SiC ceramics are crucial for radiation-resistant nuclear applications; Tynyshbayeva and Tynyshbayeva [2] recently investigated the structural disordering of SiC ceramics under heavy ion irradiation. Additionally, Lebedev et al. [3] explored the carbothermal synthesis of ultrafine SiC fibers, which is vital for developing advanced sensors and multifunctional composites. Among these diverse forms, SiCf/SiC angle-interlock (2.5D) woven composites are widely used as structural/functional components in aero-engine thermal components due to their high temperature resistance (up to 1450 °C), high out-of-plane performance, and excellent near-net-shape forming capability [4,5,6].

However, the full-scale internal spatial structure of SiC_f_/SiC (SiC fiber-reinforced SiC matrix) 2.5D woven composites is highly anisotropic and complex. It is worth noting that the pore defects are an inevitable characteristic of SiC_f_/SiC ceramic matrix composites, which can affect the prediction of the final mechanical properties and failure mechanism significantly [7,8,9]. Hence, an improved reconstructed model of SiC_f_/SiC 2.5D woven composites with pore defects is necessary for the design and construction of high-performance materials.

In a previous study, the resin matrix composites were assumed to be without pore defects [10,11,12,13]. However, the increasing demands for the refined reconstruction of composites have driven scientists to study the effects of natural defects of composites on their mechanical properties and build a precise mesoscale model with pore defects [14,15,16,17,18]. For example, Gao et al. [19] predicted the influence of pores on the mechanical properties of resin-based three-dimensional (3D) braided composites. The Mori–Tanaka method was employed to establish the constitutive model and finite element model of the 3D braided composite with pores, and simulate the evolution of the mechanical properties of the 3D braided composite with defects. A representative volume cell with pore defects of 3D five-directional braided carbon/epoxy composites was built by Ge et al. [20]. A multi-scale analysis method was established to predict the mechanical properties. The result shows that the elastic constants of 3D braided composites are significantly influenced by pores. In Chao’s study [21], a spatially random representative volume element of the microstructure carbon/carbon composite was obtained with a regular pore distribution. This method is based on an improved stochastic sequential adsorption algorithm. However, it is not hard to find that these works are aimed at the fiber reinforced resin composites where the pore volume fractions are usually less than 3%. It is worth noting that the pore defects of fiber reinforced resin composites are unnecessary meso-scale elements, while they are a crucial element for the SiC_f_/SiC textile composite due to the differences in their preparation process.

Shen et al. [22] established the representative volume element (RVE) of a 3D SiC_f_/SiC composite prepared by chemical vapor infiltration (CVI) with pores by using a scanning electron microscope. However, these pores only exist in the cross-section of the composites in the research, and the mechanical behavior of the model containing pores has not been evaluated. Few studies have taken into account the influence of pores on the mechanical properties of composites. Chateau et al. [23] investigated the influence of porosity on the mechanical properties of SiC_f_/SiC composites and the result showed that the lateral stiffness decreased to about 50% with the porosity of the composites increased from 4% to 9%. To sum up, pore defects, an important engineering defect, have a great impact on the mechanical properties of ceramic matrix composites. The current research failed to analyze the mechanical behavior based on the physical defect model of pore-containing SiC_f_/SiC 2.5D woven composites. Recent image-based and stochastic modeling studies have also incorporated pore defects in SiC/SiC composites using X-ray tomography and mesoscale FE frameworks [24,25,26,27].

In this paper, the objective is to develop and experimentally validate a full-scale finite element (FE) model that explicitly incorporates physically measured random pore defects, so as to accurately predict the bending response and damage evolution of SiC_f_/SiC 2.5D woven composites. Specifically, the statistical data of the yarns and pores of SiC_f_/SiC 2.5D woven composites are acquired by a Micro-CT experiment. Then Monte Carlo random algorithm is adopted to establish the full-scale model of SiC_f_/SiC 2.5D woven composite with pores. Furthermore, the predicted stress–deflection response, stress distribution, and damage characteristics are compared with three-point bending experiments to assess model fidelity and to demonstrate the role of pores in bending failure.

## 2. Experimental Detail

### 2.1. Materials and Specimen Preparation

**Figure 1:**
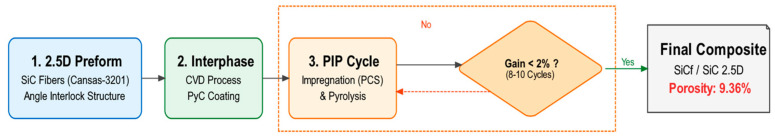
The preparation process of 2.5D woven SiC_f_/SiC composite.

The three-dimensional weave reinforcements with a layer-to-layer angle interlocking structure (2.5D) for the composites were fabricated by SiC fiber (second-generation Cansas-3201, 200 tex). A precursor immersion pyrolysis (PIP) method was conducted to prepare the SiC_f_/SiC 2.5D woven composite with two significant steps as shown in Figure 1: (1) The PyC interface layer on the surface of SiC fiber was prepared through a chemical vapor deposition (CVD) process; (2) The PIP process was employed to prepare the SiC_f_/SiC composites with precursor of the liquid polycarbosilane; (3) The immersion-cracking cycle was set as 8–10 times until the cracking weight gain rate is less than 2%. A composite material with a porosity of 9.36% was obtained eventually.

### 2.2. Three-Point Bending Tests

**Figure 2:**
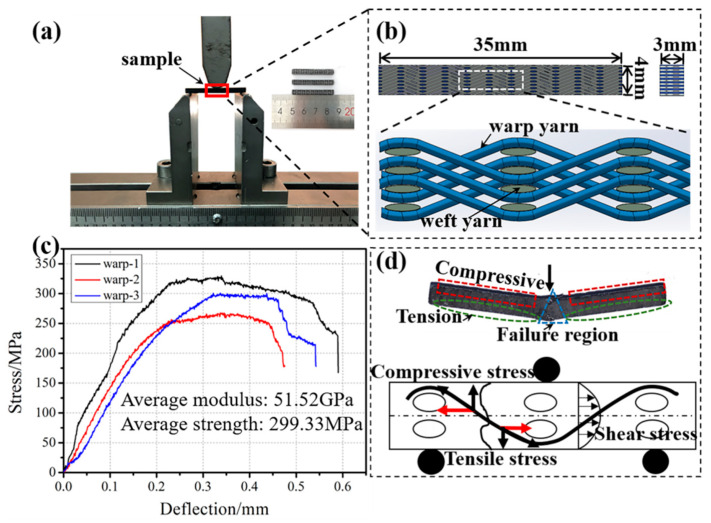
Three-point flexure setup (**a**), flexure test samples (**b**), flexure stress–deflection curves (**c**) and failure of composite (**d**). Note: Solid arrows indicate the direction of compressive/tensile stress, and hollow curved arrows represent the shear stress direction during failure.

It is well known that for 2.5D woven reinforced ceramic matrix, a relatively complex stress state with tensile, compressive and shear stresses is experienced when subjecting to bending loadings [28,29]. A number of three-point bending tests were conducted by an Instron machine (3385H load frame) at room temperature, which was equipped with a 10 kN load cell in accordance with ASTM C1341 (see Figure 2a). The three-point bending tests for all samples were performed at a crosshead speed of 0.5 mm∙min^−1^ at room temperature with a span of 30 mm. The dimensions of all specimens were 35 × 4 × 3 mm (see Figure 2b).

The stress–deflection curve obtained from the bending tests is presented in Figure 2c. It can be seen that the average bending strength of the sample is 299.33 MPa, while the average bending modulus of the sample is 51.52 GPa. Figure 2d shows the schematic diagram for the bending failure of the sample. In the bending stage, the specimen appears as compressive stress on the upper side and tensile stress on the lower side and includes in-plane shear. Here, Micro-CT (Diondo d2, diondo GmbH, Hattingen, Germany) and a super-depth three-dimensional microscope (VHX-5000, Keyence Corporation, Osaka, Japan) were adopted to observe and analyze the morphology of bending failure. A voltage of 90 kV and an accelerating current of 120 μA were used to penetrate the sample and irradiate it with attenuated intensity X-rays. Subsequently, it was received and processed on a flat panel detector with a size of 3072 × 3072 pixels with 2228 two-dimensional projection images until the sample rotated 360°. The resolution for all of the two-dimensional projections is 139 μm (22.1 μm/pixel). The three stress–deflection curves correspond to three specimens cut and tested along the warp direction (warp-1, warp-2, and warp-3), showing the experimental scatter between samples. These measured curves and the observed failure morphologies (Micro-CT and 3D microscopy) are used as the primary benchmarks to validate the proposed pore-containing FE model in Section 3 and the predictions reported in Section 4.

## 3. Establishment of Full-Scale Model of Porous 2.5D SiC_f_/SiC Composite

### 3.1. Modeling Framework

**Figure 3:**
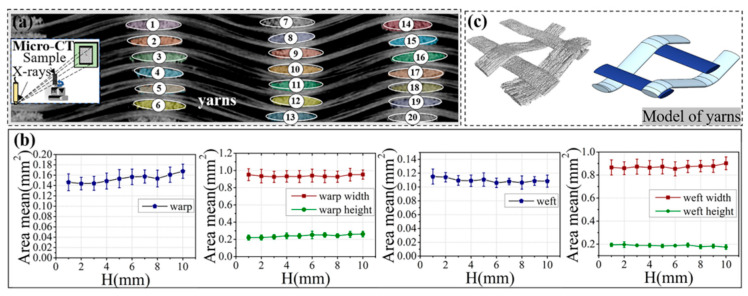
2.5D reconstruction structure of yarns: the fiber orientation of SiC_f_/SiC composite scanned by Micro-CT (**a**), the actual statistical parameters of fiber bundles (**b**), and part of yarn extraction and reconstruction (**c**). Note: Different colors and numbers in (**a**) represent the individual yarns in the 2.5D woven structure.

The geometric parameters and tomographic images were obtained from Micro-CT by following steps: (1) the VG software (VGSTUDIO MAX 3.1) was exploited to render the surface of the sample scanned by Micro-CT to make the outline of the fiber bundle clearer; (2) the cross-sectional shape of the target yarn bundles in different regions were extracted through the Image-J software (version 1.51J8) as shown in Figure 3a; (3) the statistical yarn results were numerically analyzed and exhibited in Figure 3b. Interestingly, it is found that the warp and weft cross-section parameters of the SiC_f_/SiC 2.5D woven composite exhibited good uniformity, which can provide a reliable numerical value for the assumed warp and weft parameters in the simulation model.

**Figure 4:**
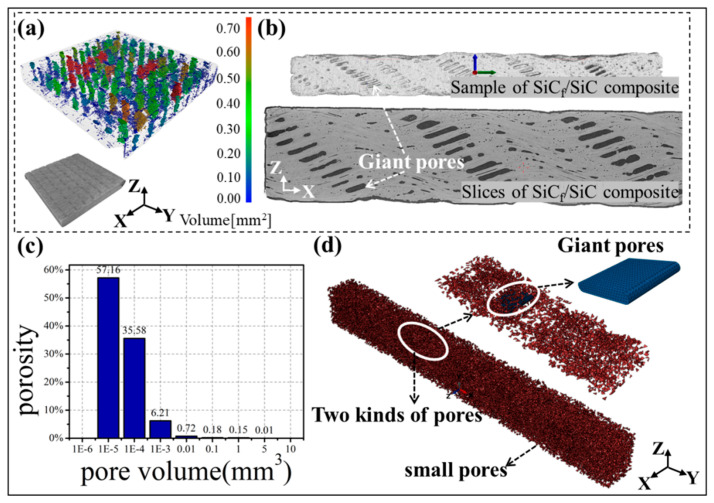
Geometrical model of SiC_f_/SiC 2.5D woven composite with pore defects: the pores of the composite extracted by Micro-CT (**a**), the distribution of giant pores in the composite (**b**), the pore distribution based on statistical results (**c**) and the classification of pores (**d**).

As mentioned above, pores will be generated in the SiC_f_/SiC composites during the preparation process, inevitably due to the restrictions of the process (Figure 4a,b). The occurrence and distribution of pore defects are closely related to the microstructure of ceramic matrix composites. In this work, we aimed at investigating the influence of pore defects on the mechanical properties of SiC_f_/SiC 2.5D woven composites. In detail, (1) The volume fraction and size of the pores in the matrix greatly exceed the pores in the fiber bundle; (2) Two main types of pores (Figure 4d) were taken into account in the model construction. Namely, giant pores under the warp yarn, and the small pores are randomly distributed. It is worth noting that the giant pores located under the warp yarns change with the position of the warp yarns; that is, the width of the giant pores is almost the same as the width of the warp yarns, which was proven by a previous study [30,31].

### 3.2. Generation of Random Pore Model

**Figure 5:**
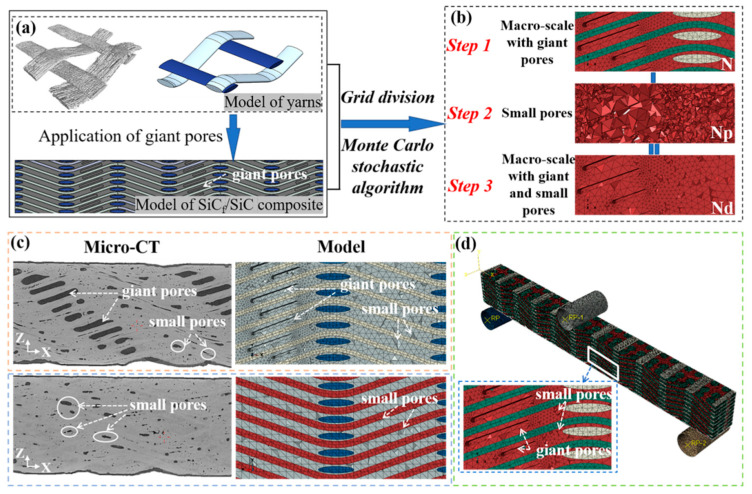
Macro structural of SiC_f_/SiC 2.5D woven composite model with pores: First, a yarn model and a giant pore model are established based on the statistical results (**a**). Then mesh the model with giant pores. Subsequently, the Monte Carlo stochastic algorithm is employed to delete the small pore units to form a finite element model (**b**). Ultimately, compare the Micro-CT scan sample with the established model (**c**). A SiC_f_/SiC 2.5D woven composite model with pores is obtained (**d**). Note: Different colors represent the yarns (red and light blue color ), matrix (gray color) in (**a**,**c**) as well as the yarns (green and light green color), matrix (red color) in (**b**,**d**), respectively.

In this work, a novel method was proposed to develop relative precise SiC_f_/SiC 2.5D woven composite model with random pores. Pore defects in the model can be divided into two groups: “giant pores under the warp yarns” and “small pores distributed randomly in the composite”. The steps for establishing a model for the composite are shown as follows: (1) A group of giant pore models was established under the warp yarns while building the yarn model according to the statistics of the “giant pores” parameters in the VG software. Those “giant pores” were eliminated then by the method of Boolean operation (Figure 5a); (2) For the group of small pores distributed randomly in the matrix, it was found that more than 80% of them had a volume of 10^−4^~10^−5^ mm^3^ based on statistical results. Hence, a Monte Carlo algorithm based on Matlab subroutines was conducted in this paper to generate small pores in the matrix randomly and to fabricate the full-scale SiC_f_/SiC 2.5D woven composite model that contains both giant pores and random small pores (Figure 5d).

The details for the novel method can be described as follows:

1. Import and discretization: Full-size SiC_f_/SiC 2.5D woven composite without giant pores created in SolidWorks is imported into ABAQUS software first, followed by grouping and meshing the yarn and the matrix. At this time, the total number of grids of the matrix is N (Figure 5b).
2. Stochastic pore assignment: A Matlab subroutine is employed to embed a random module and generate small pores in the matrix with a random distribution according to the pore content and the total number of grids. Here, the number of pores is Np (see Figure 4b).
3. Pore embedding: Generating a new matrix by deleting the number of small-pore grids in the matrix grids, and the number of grids in the matrix is noted as Nd.

Figure 5c exhibited the comparison between the partial cross-section of the composite scanned by Micro-CT and the cross-section of the established model. Which verified the accuracy of the establishment of the pore-containing model. The final 2.5D full-scale model of the SiC_f_/SiC composite with different pores can be seen in Figure 5d.

## 4. Results and Discussion

**Figure 6:**
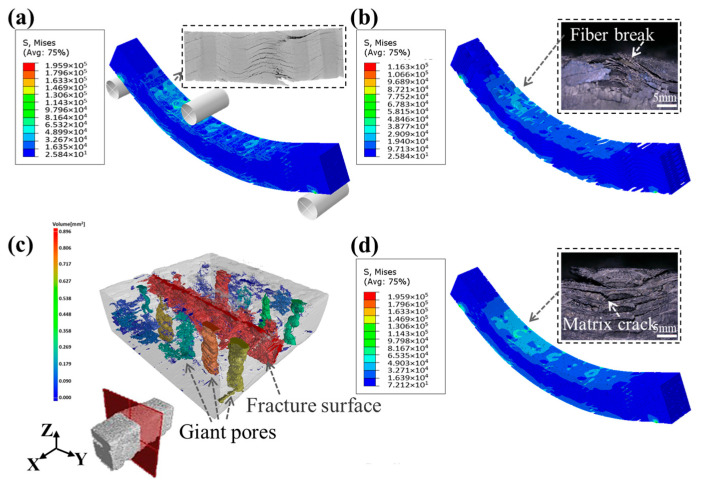
Stress nephogram of the model (**a**) and the final macroscopic failure Micro-CT diagram of the specimen (**c**) in the three-point bending test: stress nephogram of yarn (**b**), stress nephogram of matrix (**d**).

Figure 6 presents the comparison between the final damage appearance of the sample and the simulation results. It was observed that the final damage area of the sample (Figure 6c) was consistent with the simulation result (Figure 6a) and occurred at the place with “giant pores”. This is because the specimen appears as compressive stress on the upper side, tensile stress on the lower side and in-plane shear during the three-point bending test. However, the existence of pores with different sizes in the matrix has a great influence on the mechanical properties of SiC_f_/SiC composites. Resulting in the final failure of the composites expanded along the contact surface of the weft yarn and the adjacent warp yarn. And the catastrophic damage will occur around the “giant pore”, which leads to the final failure of the material.

**Figure 7:**
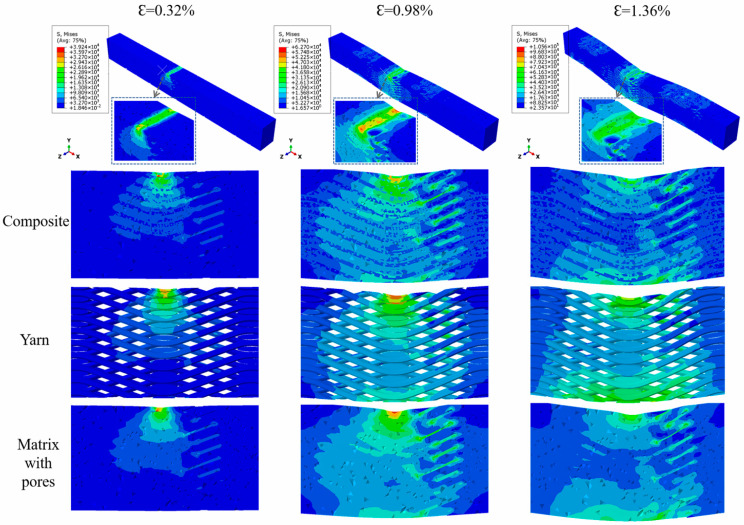
Stress nephogram of bending damage distribution of 2.5D SiC_f_/SiC composite, yarn and matrix with pores under different strains.

The damage distribution stress nephogram of the SiC_f_/SiC 2.5D woven composite with pores under three-point bending load with different strains was displayed in Figure 7 to verify the accuracy of the model. At the low strain level (elastic regime), the highest compressive stress appears beneath the upper indenter and the stress is mainly carried by the yarn architecture, with no obvious matrix cracking. At the intermediate strain level (damage initiation), local stress concentrations develop around pores and at yarn/matrix interfaces, leading to the onset of matrix cracking near the loading line and the tensile-side surface. At the high strain level (damage propagation and final failure), cracks deflect and propagate along the yarn–matrix interfaces and around the pore clusters, and the damaged zone extends toward the indenter while tensile-side cracking becomes more pronounced. The progressive crack deflection/bridging and frictional sliding at the PyC interphase contribute to the observed pseudo-plastic bending response in the experiments (Figure 2c).

## 5. Conclusions

In this work, we originally propose a full-scale model for the mechanical behavior in bend stages for the SiC_f_/SiC 2.5D woven composites with random pore defects. The three-point bending experiment is conducted to validate the proposed model. The main conclusions can be summarized as follows:

1. The 2.5D woven SiC_f_/SiC composite model constructed based on statistical analysis and Monte Carlo random function is in good agreement with the mechanical damage and failure behaviors in the experiment.
2. The existence and size of the pores have a great impact on the mechanical properties of SiC_f_/SiC 2.5D woven composites. It was observed that the final failure of the SiC_f_/SiC 2.5D woven composite is to expand along the contact surface of the weft yarn and the adjacent warp yarn, and catastrophic damage occurs in the “giant pores”, which leads to the final failure of the material.

The results demonstrate that the proposed defect model with random pores can describe the mechanical damage behavior of SiC_f_/SiC 2.5D woven composites effectively. The simulation results yielded a bending strength that deviates by less than 5% from the experimental average (299.33 MPa), validating the accuracy of the random pore model. It may provide an idea and guidance for developing a precise model for simulating the damage process of the SiC_f_/SiC 2.5D woven composites.

## Data Availability

The original contributions presented in this study are included in the article. Further inquiries can be directed to the corresponding authors.

## Abbreviations

The following abbreviations are used in this manuscript:

SiC_f_/SiC	SiC fiber-reinforced SiC matrix
2.5D	Angle-interlock
PIP	Precursor immersion pyrolysis
FE	Finite element
Micro-CT	Micro-computed tomography
CVD	chemical vapor deposition
